# Additivity and Interactions in Ecotoxicity of Pollutant Mixtures: Some Patterns, Conclusions, and Open Questions

**DOI:** 10.3390/toxics3040342

**Published:** 2015-09-25

**Authors:** Ismael Rodea-Palomares, Miguel González-Pleiter, Keila Martín-Betancor, Roberto Rosal, Francisca Fernández-Piñas

**Affiliations:** 1 Departamento de Biología, Facultad de Ciencias, Universidad Autónoma de Madrid, Madrid E-28049, Spain; E-Mails: ismael.rodea@uam.es (I.R.-P.); mig.gonzalez@uam.es (M.G.-P.); keila.martin@uam.es (K.M.-B.); 2 Departamento de Ingeniería Química, Universidad de Alcalá, Alcalá de Henares, Madrid E-28871, Spain; E-Mail: roberto.rosal@uah.es

**Keywords:** mixtures, additivity, synergism

## Abstract

Understanding the effects of exposure to chemical mixtures is a common goal of pharmacology and ecotoxicology. In risk assessment-oriented ecotoxicology, defining the scope of application of additivity models has received utmost attention in the last 20 years, since they potentially allow one to predict the effect of any chemical mixture relying on individual chemical information only. The gold standard for additivity in ecotoxicology has demonstrated to be *Loewe additivity* which originated the so-called Concentration Addition (CA) additivity model. In pharmacology, the search for interactions or deviations from additivity (synergism and antagonism) has similarly captured the attention of researchers over the last 20 years and has resulted in the definition and application of the Combination Index (CI) Theorem. CI is based on Loewe additivity, but focused on the identification and quantification of synergism and antagonism. Despite additive models demonstrating a surprisingly good predictive power in chemical mixture risk assessment, concerns still exist due to the occurrence of unpredictable synergism or antagonism in certain experimental situations. In the present work, we summarize the parallel history of development of CA, IA, and CI models. We also summarize the applicability of these concepts in ecotoxicology and how their information may be integrated, as well as the possibility of prediction of synergism. Inside the box, the main question remaining is whether it is worthy to consider departures from additivity in mixture risk assessment and how to predict interactions among certain mixture components. Outside the box, the main question is whether the results observed under the experimental constraints imposed by fractional approaches are a *de fide* reflection of what it would be expected from chemical mixtures in real world circumstances.

## 1. Introduction and Aims

What is the effect of pollution on human health and the environment under realistic conditions? Realistic conditions imply several layers of complexity which complicate problem definition and hypothesis testing. The mixture aspect is intrinsic to environmental pollution; however, the implications of the “mixture” aspects, the experimental methods to study them, and their implications for risk assessment are still under intense investigation [1,2,3,4]. Presently, the gold standard supporting mixture effect research and mixture risk assessment in human toxicology and ecotoxicology are fractional additivity methods (CA, IA, CI) [5,6]. Derived from human toxicology, additivity methods are based on the basic idea that the expected effect of a mixture of chemicals can be predicted based on the sum of the fractions of effects of the individual mixture components. Departures from additivity (synergism and antagonism) are defined as deviations from this behavior [5,6]. With the present critical review, we revised the recent history of development and applications of the fractional additivity models CA, IA, and CI (Section 2 and Section 3), culminating with the latest European Legislation on mixtures [7]. Despite there exists a robust body of experimental laboratory evidence indicating that additivity models offer a reasonable expectation of mixture toxicity, the possibility of the occurrence of unpredictable departures from additivity, and especially synergism, is a non-solved problem. Therefore, we formally address the evidence suggesting the possibility of predicting interactions and specifically synergistic effects among mixture components (Section 4). Finally, we tried to raise a series of questions regarding the actual applicability of fractional additivity models in conditions of increasing realism and, therefore, complexity (Section 5). The aim of the section was not just to say this or that study was in agreement or not with the additivity prediction. Our aim was to reflect on the theoretical and experimental constraints of the fractional approaches and how these constraints determine both our understanding of the “mixture” problem, and the practical applicability of fractional approaches in the increasing complexity implied by the urged increasing realism which has been established as a central milestone in the new ecotoxicological agenda for the 21th Century [2].

## 2. Additivity and Deviations from Additivity

The mixtures of pharmaceuticals have been of medical concern since ancient times. Drugs which act similarly are often combined to treat a number of diseases as synergistic effects are usually expected. Synergistic drug combinations allow to deliver lower doses to the patient, minimizing adverse effects. In this context, there is a need to define synergism and its opposite, antagonism, which are considered as toxicological interactions in chemical mixtures. There are many definitions of both terms as well as different synonyms. Goldin and Mantel [8] collected up to seven definitions of synergism with synonyms such as synergy, potentiation, augmentation, sensitization, suppraadditiveness, superadditivity, potentiated summation or positive summation. For antagonisms, terms such as depotentiation, desensitization, infraadditiveness, subadditivity, negative synergy, *etc.*, have been used. Therefore, there was a need for consistent and clear terminology to describe toxicological interactions; in this regard, EPA [9], Mumtaz and Hertzberg [10], Hertzberg *et al.* [11], EPA [12], and ATSDR [13] provided distinct definitions for some of these terms: no apparent influence (“when a component which is not toxic to a particular organ system does not influence the toxicity of a second component on that organ system”); synergism (“when the effect of the mixture is greater than that estimated for additivity on the basis of the toxicities of the components”); potentiation (“when a component that does not have a toxic effect on an organ system increases the effect of a second chemical on that organ system”); antagonism (“when the effect of the mixture is less than that estimated for additivity on the basis of the toxicities of the components”); inhibition (“when a component that does not have a toxic effect on a certain organ system decreases the apparent effect of a second chemical on that organ system”), and masking (“when the components produce opposite or functionally competing effects on the same organ system, and diminish the effects of each other, or one overrides the effect of the other”).

In the context of the above definitions, toxicological interactions such as synergism and antagonism may be considered as departures from additivity. Berenbaum [14] already described synergism as the situation where the effect of a drug combination is greater than expected, and antagonism as the situation in which the combination response is less than expected and no or zero interaction as the situation where the drug combination effect is as expected. He also referred to zero interaction as additivity but advised that the term does not imply anything about how the operation of addition is performed. Goldin and Mantel [8] described synergism or antagonism when the potency of the mixture/combination is greater or less than can be accounted by the potencies of the individual drugs. When the combination effect is as expected (consistent with the individual drug potencies), the interaction is then additive.

As stated by Tallarida [15] and many others, the concept of additivity in drug mixtures is not the simple addition of effects; so that the key question is what is the definition of addivity, zero interaction, or “as expected”, as this provides the basis for the assessment of synergism and antagonism. A frame/model for addivity, zero interaction, or “as expected”, is needed. The EPA [12] defined additivity as the situation “when the effect of the combination is estimated by the sum of the exposure levels or the effects of the individual chemicals. The terms ‘effect’ and ‘sum’ must be explicitly defined. Effect may refer to the measured response or the incidence of adversely affected organisms. The sum may be a weighted sum (see ‘dose addition’) or a conditional sum (see ‘response addition’)”. Therefore, there are two mathematical models widely accepted in pharmacology and later in ecotoxicology which are the so-called dose addition (similar joint action) which applies to mixtures of drugs with the same mode of action and response addition (independent joint action) which applies to mixtures of drugs with different modes of action [16,17,18,19]. Concentration Addition (CA) or Loewe additivity are synonyms of dose addition, while Independent Action (IA) or Bliss independence are synonymous to response addition.

Compounds with similar modes of action are expected to be mutually exclusive and behave as higher doses of a single compound. Dose addition, or Concentration Addition (CA), is then based on the assumption that all components in the mixture behave as if they are simple dilutions of one another [20]. For a mixture of “*n*” components, the CA concept can be expressed as follows:

(1) $$\frac{\sum_{i=1}^{n}d_{i}}{D_{x,mix}}\;=\;\frac{c_{mix}}{D_{x,mix}}\;=\sum_{i=1}^{n}\frac{d_{i}}{D_{x,i}}$$

where *d_i_* is the dose for chemical “*i*” in the mixture, *D_x_*_,*i*_ the concentration of the “*i*” component that causes “*x*” effect for a given endpoint when individually exposed, *c_mix_* the total concentration of the mixture, and *D_x_*_,*mix*_ the mixture concentration causing the same effect “*x*”. As *c_mix_* = *D_x_*_,*mix*_ the well-known form for CA arises:

(2) $$\sum_{i=1}^{n}\frac{d_{i}}{D_{x,i}}\;=\;1$$

It is important to point out that, under the model of CA, mixture components below their individual no-effect concentration (NOEC) may nevertheless contribute to the total effect of the mixture.

In contrast, response addition or Independent Action (IA) applies to chemicals with dissimilar modes of action [16]. IA assumes that the mixture components cause a common effect through primary interaction with different target sites [21]. It is the case of mutually non-exclusive drugs for which the effect (response), and not the dose, behaves additively. As IA is based on effects, a relevant difference with the CA model is that a mixture component used in a concentration below its NOEC will not contribute to the total effect of the mixture. The mathematical form is as follows:

(3) $$D_{x,mix}\;=\;1\;-\;\prod_{i=1}^{n}\left(1\;-\;D_{x,i}\right)$$

**Figure 1:**
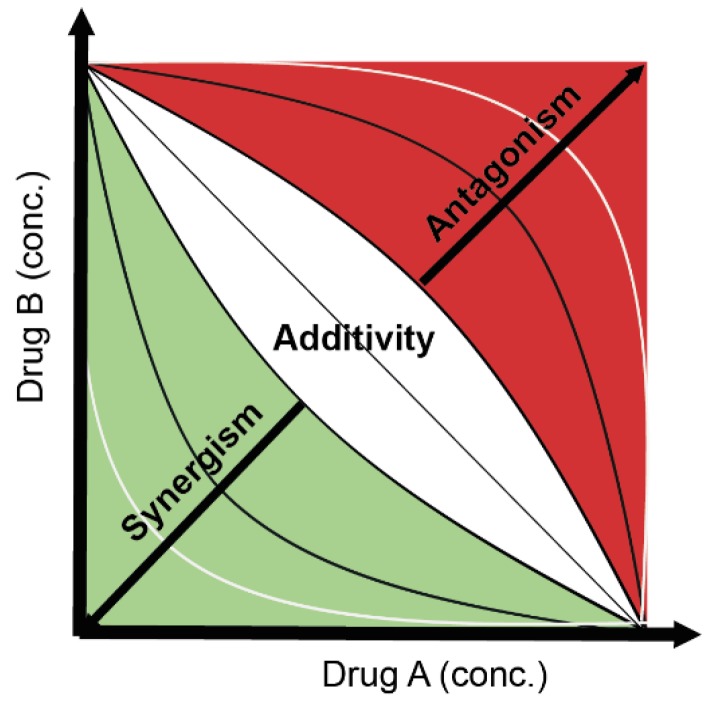
Schematic isobologram showing the equi-effective Cartesian plane for the combination of Drug A and Drug B at a specific effect level *fa* (for example *fa* = 0.5). For any mixture ratio (A:B), the effect of A + B should be theoretically located on the straight transversal line (the additivity line). The additivity effect can consider uncertainty (depicted by the white region). When the doses of A + B required to get the desired effect (*fa*) is less than that expected from their theoretical fractional summation of their respective individual effects (see Equation (4)), the effect of A + B is synergistic, and their coordinate location in the isobologram Cartesian plane is below the additivity line, depicting synergism. The opposite case indicates antagonism. Figure adapted from Cokol *et al.* [25]. Copyright 2011, EMBO and Macmillan Publishers Limited.

Fraser [22] was the first to introduce the isobole method to study drug combinations in the field of pharmacology. He used this approach to study drug antagonisms but it serves as a good starting frame to study departures from additivity [19,23,24]. The isobole is a graphical method which shows dose combinations of two drugs that yield the same effect; it is a Cartesian coordinate system in which doses of two drugs are represented in the *x*- and *y*-axes (Figure 1). Isobolgram can be mathematically expressed as follows (for notation see Equation (1)):

(4) $$\frac{d_{1}}{D_{1}}+\frac{d_{2}}{D_{2}}=1$$

If for a certain effect level, *i.e*., 50%, there is no interaction between the two drugs in combination, a straight line connects the intercepts (doses) in the *x*- and *y*-axes (isobologram equation = 1) (Figure 1). However, when the line connecting both doses lies below and to the left of the line of additivity (concave-up line), synergism is found (isobologram equation <1). When the line connecting both doses lies above and to the right of the line of additivity (concave-down), antagonism is found (isobologram equation >1) (Figure 1).

The isologram equation may be generalized to *n* drugs as a sum of fractions:

(5) $$\frac{d_{1}}{D_{1}}+\frac{d_{2}}{D_{2}}......+\frac{d_{n}}{D_{n}}=1$$

The sum of all fractions has been termed as the Interaction Index [14] and gives a measure of the degree of synergism or antagonism although it is not clear whether these deviations are statistically significant or not. (Note that Equation (5) is in fact equivalent to Equation (2) but derived from the isobologram definition).

In an effort to evaluate the statistical significance of deviation from the additivity line, Syberg *et al.* [26] added an extra parameter to the isobole formula, denoted as λ which described the degree of concavity of the isobole reflecting the degree of synergism/antagonism. In this regard, Altenburger [27] already suggested the use of confidence intervals for the additivity line in order to determine whether true synergism or antagonism held; however, they also pointed out than an estimate of the confidence interval for the mixture should be given to further support the observed interaction.

The isobole is however a graphic method and it seems obvious that with more than 3 components (three-dimensional isobole surface), the graphic is difficult to make and visualization of the effect of mixtures is difficult [14,27]. It is also important to apply the method to as many effect levels as possible as departures from additivity might change with different effect levels. Usually, isoboles are drawn for the 50% effect level. An advanced isobologram representation for extensive data generation as a function of mixture ratio can be found in Cokol *et al.* [25].

Isoboles are generally based on the CA model, however, Berembaum [14] and Syberg *et al.* [26] successfully applied isoboles based on the theory of IA. In fact, as defined by Berenbaum [14], isoboles do not depend on mechanistic assumptions and are independent of the shapes of the dose-response curves so that they might apply for both CA and IA models of additivity.

Further extension of the isobole method is the Isobologram-Combination Index developed by Chou and Talalay [28,29,30], Chou and Martin [31], and Chou [5]. The Combination Index (CI) equation/theorem, also known as the multiple drug-effect equation, was derived from the median-effect principle of the mass-action law [30]. The medium-effect principle or equation is also called the general theory of dose and effect that demonstrates that there is a univocal relationship between dose and effect independently of the number of substrates or products and of the mechanism of action or inhibition [5]. Chou and coworkers, after years of progression and development, formulated the general median-effect Equation (6) which they claim as a unified theory (derived from over 300 mechanistically distinct equations of biomedical sciences) of dose and effect which linearizes dose-effect curves with different potencies and shapes. This equation is a simple way to relate dose and effect showing that dose and effect are *de facto* interchangeable:

(6) $$\frac{fa}{fu}={\left(\frac{D}{D_{m}}\right)}^{m}$$

where *D* is the dose, *D_m_* is the dose for 50% effect, *fa* is the fraction affected by dose *D*, *fu* is the unaffected fraction (*fa* = 1 − *fu*), and *m* is the slope of the dose-effect curve which depicts the shape of the curve (*m* = 1, >1, and <1 indicate hyperbolic, sigmoidal, and flat sigmoidal curve, respectively). Therefore, the method takes into account both the potency (*D_m_*) and slope (*m*) parameters. If Equation (6) is rearranged, then:

*D* = *D_m_*·[*fa*/(1 − *fa*)]^1/*m*^ (7)

The *D_m_* and *m* values for each drug are easily determined by the median-effect plot: *x* = log (*D*) *versus* *y* = log (*fa*/*fu*) which is based on the logarithmic form of Equation (7). In the median-effect plot, *m* is the slope and *D_m_* is the median-effect dose (antilog of the *x*-intercept of the median effect plot). The conformity of the data to the median-effect principle can be ready manifested by the linear correlation coefficient (*r*) of the data to the logarithmic form of Equation (7) [5].

Based on the general median-effect equation, Chou and Talalay [29,32] developed the CI equation for the quantification of synergism or antagonism for *n*-drug combination at *x*% inhibition:

(8) $${}^{n}{\left(CI\right)}_{x}=\sum_{j=1}^{n}\frac{{\left(D\right)}_{j}}{{\left(D_{x}\right)}_{j}}=\sum_{j=1}^{n}\frac{{\left(D_{x}\right)}_{1-n}\left\{{\left[D\right]}_{j}/\sum_{1}^{n}\left[D\right]\right\}}{{\left(D_{m}\right)}_{j}{\left\{{\left(f_{ax}\right)}_{j}/\left[1-{\left(f_{ax}\right)}_{j}\right]\right\}}^{1/mj}}$$

where ^*n*^(CI)_*x*_ is the combination index for *n* drugs at *x*% inhibition; (*D_x_*)_1−*n*_ is the sum of the dose of *n* chemicals that exerts *x*% inhibition in combination, $D_{j}/\sum_{1}^{n}\left[D\right]$ is the proportionality of the dose of each of *n* chemicals that exerts *x*% inhibition in combination; and (*D_m_*)_j_·{(*f_ax_*)*_j_*/[[1 − (f_ax_)_j_]}^1/*mj*^ is the dose of each drug alone that exerts *x*% inhibition. From Equation (8), CI < 1, CI = 1, and CI > 1 indicates synergism, additive effect, and antagonism, respectively.

**Figure 2:**
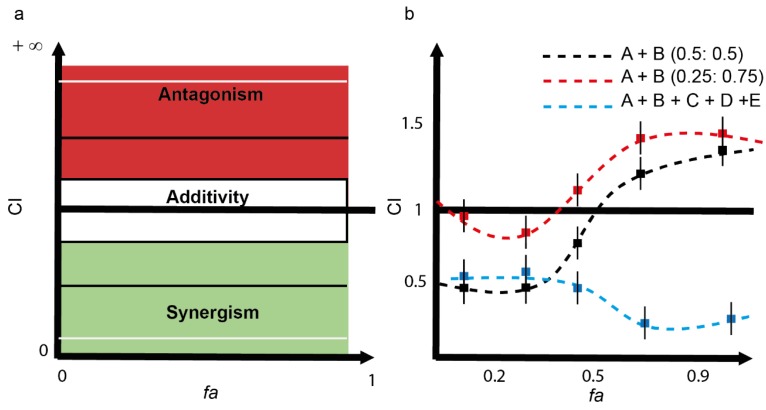
(**a**) Schematic representation of a *fa-*CI plot. In contrast to the Isobologram, *fa*-CI plots are effect-oriented graphics (see *x*-axis). The additivity line is CI = 1; CI < 1 indicates synergism and CI > 1 indicates antagonism. The additivity effect can consider uncertainty (depicted by the white area). The type of combined effect between any combinations of drugs (from 2 to *n*) can be shown for the entire range of fractional effect levels (from 0 to 1) without the requirement of extra graphical dimensions; and (**b**) schematic representation of three theoretical mixtures, in the same graphic, the effect-dependent type of interaction of drugs A + B can be visualized for the same mixture (A + B) at two different mixture ratios (0.5:0.5) and (0.25:0.75). In addition, the effect-dependent interaction of a hypothetical complex mixture composed of A + B + C + D + E can be similarly visualized. For experimental examples, see references [33,34,35,36,37,38].

Software CompuSyn [31] (Combosyn Inc., Paramus, NJ, USA) was developed for calculation of dose-effect curve parameters, CI values, conventional isobolograms, *fa*-CI plot (plot representing CI *versus* *fa*, the fraction affected by a particular dose; see Equation (8)), and polygonograms (a polygonal graphic representation depicting synergism, additive effect and antagonism for three or more drug combinations). The *fa*-CI plot, as an effect-oriented graphic, complements the isobole, which is a dose-oriented graphic and allows showing the interaction between drugs in a combination at all effect levels simultaneously. Figure 2a shows the schematic representation of the location of additivity, synergism, and antagonism in the *fa*-CI plot. Different mixture ratios, different binary mixtures or multicomponent mixtures without any limitation regarding the number of components can be drawn in the *fa*-CI plot (Figure 2b).

**Figure 3:**
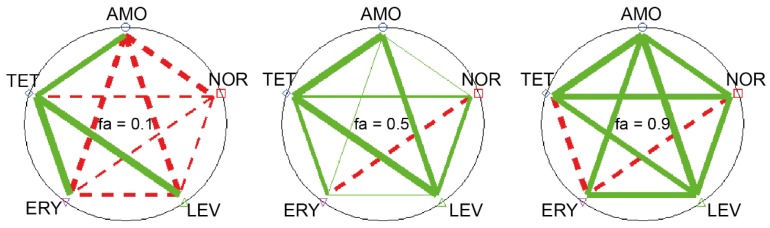
Polygonograms showing pair-interactions among 5 antibiotics for three selected effect levels, *fa* (0.1, 0.5, and 0.9) in the bioluminescent cyanobacterium *Anabaena* CPB4337 (elaborated based on the data published in [38]). AMO: Amoxicillin; NOR: Norfloxacin; LEV: Levofloxacin; ERY: Erythromycin; TET: Tetracycline. Green lines denote synergism and red lines antagonism. The thickness of the lines indicates the strength of the interactions, (thickness scales according to [5]). The polygonogram allows an effect-oriented visualization of the nature of the combination effect of mixtures. Here binary mixtures (line connectors) are presented. However higher than two component interactions can also be graphically presented. For example, ternary mixture interactions can be represented showing triangles among three chemicals, quaternary by squares, *etc*.

As an example of the polygonogram semi-quantitative representation of drug-drug interaction, binary interactions among five antibiotics of different families are presented for three fractional effect (*fa*) levels (0.1, 0.5, and 0.9) in Figure 3. As can be seen in Figure 3, polygonograms are a useful tool to analyze pair-pair interactions and global tendencies in an effect oriented configuration. For example, from Figure 3 it can be seen that tetracycline and levofloxacin, and tetracycline and norfloxacin generated synergistic interactions independently of the effect level analyzed, while tetracycline and erythromycin resulted in effect level-dependent interactions (from synergistic at low effect levels (*fa* = 0.1) to antagonism at high effect levels (*fa* = 0.9).

As in both, the median-effect and CI equations, each term is a ratio, units are dimensionless which means that in any drug combination, each drug may be in different units (M, mg/L, *etc.*); even a drug *per se* might already be a mixture. An interesting feature of the method is that no previous knowledge about chemical structures or modes of action are needed to quantify the potential interactions between drugs.

## 3. Concentration Addition and Independent Action in Ecotoxicology

From the pioneering work of Hermens*, et al.* [39], the idea of using the two simple pharmacological concepts of concentration addition (CA) and independent action (IA) as reasonable references to predict combined effects of chemical mixtures gained strong interest in ecotoxicology. This pioneering work, and the follow ups of Faust, Altenburger, Backhaus and co-workers [21,40,41,42,43,44,45,46], were motivated by a lack of a reference framework for mixture research in ecotoxicology which resulted in a general perception of unpredictability of mixture expected toxicity [1]. From 1999, a series of well-designed experiments were planned and developed [40,41,42,46] with three main objectives: (1) to test whether the two existing pharmacological definitions of additivity (CA and IA) were able to offer a reasonable quantitative estimation of the experimental toxicity produced by multicomponent mixtures; (2) to test whether the effects of mixtures of purely similarly and dissimilarly acting chemicals were predicted better by CA and IA additivity models, respectively; and (3) to test whether or not toxicants near or below the statistically significant effect limit contributed to the resulting toxicity of the mixtures. This series of experiments revealed important key aspects of the ecotoxicity of pollutant mixtures which changed and defined the subsequent 20 years of mixture research in ecotoxicology [1]. Regarding the first question, CA and IA showed an unexpected strong predictive power of the toxicity of multicomponent mixtures towards relevant model organisms. In general, the experimental toxicity of pollutant mixtures deviated less than a factor of two of the predictions based on the reference models (CA and IA) [21,40,41,42]. In fact, it was demonstrated that IA prediction always underestimated CA predictions and never deviated from CA more than a factor of *n* (the number of components in the mixtures) [21]. Therefore, CA (Loewe additivity) was later proposed as the gold standard for additivity [6]. Regarding the second question, the initial experiments with multicomponent mixtures of Backhaus *et al.* [41] with purely similarly acting chemicals (herbicides from the family of PSII inhibitors), and purely dissimilarly acting chemicals [40] were more acutely predicted by CA (model for similarly acting chemicals) and IA (model for dissimilarly acting chemicals), respectively. Despite these first insights on the applicability of both models, further research on the goodness of the fit of CA and IA revealed that the criterion on similar/dissimilar modes of action for the selection of the additivity model was not so consistent regarding other model organisms [47]. As reported, some pollutants may present different and unknown modes of action on non-target organisms, such as for example herbicides on amphibians [48]. This is especially true for pharmaceutically-active chemicals whose pharmacological mode of action on humans or mammals may be well understood, but whose mechanisms of toxic action on non-target species is generally largely unknown [34,47]. Regarding the third point: whether or not toxicants at concentrations near or below their statistically significant effect may contribute to the resulting toxicity of the mixtures; the follow up experiments designed by Junghans *et al.* [21], Faust *et al.* [42],Walter *et al.* [46] were clearly oriented to answer this question: In Faust *et al.* [42], 18 purely similarly-acting chemicals (s-triazines) were mixed at their no observed effect concentrations (NOECs) and checked for their combined toxicity to the green alga *Scenedesmus* *vacuolatus*. Similarly, Walter *et al.* [46] explored the combined toxicity of 11 totally dissimilarly-acting chemicals towards *Scenedesmus* *vacuolatus*. Interestingly, these works clearly demonstrated that, independently of similar or dissimilar modes of action (CA and IA models, respectively), a mixture of substances below their NOEC was able to produce greater toxicity than any of the individual chemicals, implying that predictions from additivity models, such as IA, are not always right. In fact, those experiments were a clear proof of the “nothing to something” concept, which was strikingly surprising at that time [1]. In addition, the resulting mixture toxicity was accurately predicted by the CA model for similarly acting chemicals, and by IA model for the purely dissimilarly acting chemicals. The last result was unexpected and outmost interesting at this time since the emergence of toxicity in mixtures of purely dissimilarly acting chemicals below their individual NOECs [42,46] was non-consistent with the theory behind the IA model in the sense that the multiplication of zero effects remains zero (see Equation (3)), (note that the effect multiplication in Walter *et al.* [46] was performed with the interpolated theoretical effects from the dose-response curves at the NOEC doses for each individual chemical). After these initial experiments, CA and IA models became more and more popular in ecotoxicology as both theoretical and practical framework which have defined the present foundations of mixture ecotoxicology and mixture risk assessment [6]. In fact, the empirical evidences of the mixture effects uncovered by the individual chemical by chemical investigation was so strong that they forced regulatory entities at the UE level to start a fruitful dialogue and subsequent regulatory development incorporating, explicitly, mixture risk assessment in regulatory frameworks (Mixture Conclusion of the Council of European Ministers in 2009) [49], the state of the art on mixture toxicity [6], and the opinion by the European Scientific Committees [7]. These regulatory efforts culminated in the communication from the commission on combination effects of chemicals (COM/2012/0252 final) at the European level, and with the incorporation of specific methods for environmental risk assessment (ERA) of chemical mixtures in legislations of different national and international organizations (WHO IPCS programs) [50].

## 4. Combination Index in Ecotoxicology

Rodea-Palomares and co-workers [33,34,35,38,51] were the first to apply the Combination Index (CI)-Isobologram Equation to study the toxicological interactions of priority and emerging pollutants in an ecotoxicological context (using several aquatic organisms). They tested heavy metals, as well as emerging pollutants (lipid regulators, chlorinated pollutants, per-fluorinated surfactants and antibiotics) and environmental samples, namely wastewaters, in binary and complex mixtures. As model organisms they used a battery of aquatic organisms, including a natural bioluminescent organism (*Aliivibrio* *fischeri*), the recombinant bioluminescent cyanobacterium *Anabaena* CPB4337, the crustacean *Daphnia magna*, and the green alga *Pseudokirchneriella subcapitata*. Some of their most interesting findings are that the nature of the interaction between chemicals is strongly dependent on the effect level exerted by the mixture on the organism, so that the same pollutants can act synergistically at low effect levels and antagonistically at high effect levels [33,34,51]. They also found that emerging pollutants with the same pharmacological mode of action (such as fibrates or antibiotics) can strongly interact synergistically in non-target organisms by unknown toxicological modes of action [34], and that the nature of the interaction between chemicals is strongly dependent on the test species [35]. They also found that a complex mixture comprising pharmaceuticals and a wastewater from a local treatment plant with more than 30 micropollutants detected interacted in a synergistic way at all effect levels [34]. This finding is interesting in the context of increasing realism in mixture ecotoxicological research (see following sections). They proposed that the CI method can be applied in environmental toxicology as a general method to define interactions of potential toxicants in mixtures toward target and non-target organisms. This is since no previous knowledge on the pharmacological/toxic mechanism of action of the pollutants is needed and that the method may be especially useful for risk assessment strategies that take into account the toxicological interactions of substances in a mixture. In addition, the CI method offers several graphical representations which have increased power with respect to the classical isobologram method: the *fa-*CI plots and the polygonogram plots [5] (see previous sections). As stated, *fa-*CI plots and the polygonograms are effect-oriented (in contrast to isobolograms, which are dose-oriented). In addition, effect-oriented interactions can be represented for mixtures of any number of components. This attributes are particularly useful for the identification of effect level-dependent departures from additivity, and especially to identify synergistic interactions at low/very low effect levels of pollutants [34]. Recently, Gonzalez-Pleiter *et al.* [38] performed a study on the toxicity of complex mixtures of antibiotics finding that classical CA and IA predictions underestimated the toxicity of antibiotics. In addition, a predictive equation based on CI equation was developed in this work. The method, which incorporated the CI values derived from experimental evaluation of mixture toxicity was shown to predict more accurately than CA and IA the toxicity of mixtures of antibiotics. The exercise was only demonstrative, since to truly use CI as a predictive method, it is required to assign a numerical value to CI. The problem therefore is how to predict the CI values for a specific mixture of chemicals. For the moment, it has not been addressed satisfactorily.

Boltes *et al.* [52] used CI to study the toxicity of mixtures of PFOS (perfluorooctane sulphonic acid) with chlorinated chemicals (triclosan and 2,4,6-trichlorophenol) and lipid regulators (two fibrates) towards *P. subcapitata*. They found that PFOS modified the toxicity of all tested compounds independently of their individual toxicity and, most interestingly they found that systematically all the ternary mixtures were more synergistic than their binaries, which again agrees with the notion that the complexity of the mixture tends to increase synergism.

Carvalho *et al.* [53] also used the CI approach to study the toxicological interactions of 8 preservatives in binary mixtures with an industrial wastewater towards the bacteria *A. fischeri*, *Pseudomonas putida*, the protozoan *Tetrahymena thermophila* and activated sludge. Nine of the binary combinations showed significant interactions as quantified by CI which were under or overestimated by the CA model. Interestingly, the IA model showed a higher predictive power than CA in the biological community of activated sludge.

Recently, Wang *et al.* [36,37] and Chen *et al.* [54,55] have applied the CI to study the combined toxicity of insecticides, herbicides, and cadmium to a terrestrial organism, the earthworm *Eisenia fetida*. In general, their finding are similar to those of Rodea-Palomares and co-workers (see above) in aquatic organisms. Chen *et al.* [54], following the predictive rearrangement of the CI equation developed byGonzalez-Pleiter *et al.* [38] found that synergism increased with the complexity of the mixture with CI as the method predicting more accurately the combined toxicity.

## 5. Is It Possible to Predict Synergism?

Additivity assumption is very convenient for risk assessment purposes since it allows the direct computation of joint effect of chemical mixtures relaying on single component information [1,6]. The main practical disadvantage of considering departures from additivity in a risk assessment framework is the explicit requirement of correcting additivity predictions [56]. Since interactions are, in principle, non-predictable, it forces to perform these corrections on a case-by-case basis. In addition to the practical issues, more than additive effects (synergism) has been considered scarce in the ecotoxicological context based on experimental results obtained mainly in pesticide research during the last 15 years [1,6,57]. However, a recent, thorough revision of available ecotoxicological literature on the prevalence of synergism, despite confirming that synergistic interactions are actually scarce in the pesticide context (a 5% incidence), evidence that they are more abundant in other contexts, such as in biocide formulations (26% of incidence) [58].

Therefore, the possibility of predicting the occurrence of non-additive interactions (especially synergistic ones), may be justified and may appear attractive in such contexts. The potential prediction of non-additive interactions can be set at two potential levels: (1) the prediction of synergism (or antagonism) among pairs of chemicals, based on specific chemical or biological information; and (2) the prediction of multicomponent interactions based on previous knowledge on component-component interactions.

### 5.1. Prediction of Synergism (or Antagonism) among Pairs of Chemicals

Regarding the first possibility, the detection and prediction of drug-drug interactions *in silico* is in fact an open challenge in toxicology and ecotoxicology [59,60]. It is clear, however, that if the issue should be addressed, only a systems approach appears as a tractable way forward [60]. Ecotoxicology is presently, in our opinion, far from having an available systems approach suited to support an investigation of *in silico* prediction of synergistic effects among pairs of chemicals. In fact, even in pharmacology, only recently the *in silico* prediction of synergism has been really undertaken in a systematic way [59]. The work reported by Bansal *et al.* [59] is a clear example on how new open crowdsourcing collaborative research may add disruptive value to solve historically complicated problems. The DREAM consortium (Dialogue for Reverse Engineering Assessments and Methods) is a non-profit international organization devoted to identify and manage computational challenges (at present related with human health). Bansal *et al.* [59] reported the result of the challenge entitled “DREAM7-NCI-DREAM, Drug Sensitivity and Drug Synergy Challenge” [61]. In this initiative, computational scientists were challenged to rank 91 compound pairs, from the most synergistic to the most antagonistic based on gene-expression profiles of human B cells exposed to individual compounds at multiple time points and concentrations. The challenge resulted in 31 different community generated computational approaches that intended to predict synergistic pairs. The criteria to define additivity, synergism and antagonism was Bliss additivity (IA) (no clear justification was offered for this decision in the paper). The models developed considered a variety of hypotheses and information sources to build up the predictions. The ideas underlying the best performing models are quite interesting: the first ranked model, DIGRE (drug-induced genomic residual effect) hypothesized that when cells are sequentially treated with two compounds, the transcriptional changes induced by the first drug contribute to the effect of the second. The algorithm models synergism sequentially (despite experimental exposure was not). They overlapped differentially-expressed genes after treatment with the two compounds, and computed a similarity score. The key idea in the computation of synergism is the concept of “compound induced transcriptomic residual effects” which means that the synergism of two compounds depends, at least partially, on the transcriptomic changes induced by one chemical which contributes to toxicity after the treatment with the other. The hypothesis underlying the second best performing method called IUPUI_CCB is also quite interesting: it hypothesized that the activity of a compound can be predicted from its effect on the genes that are significantly differentially-expressed following treatment with highly toxic compounds *versus* control media. Synergism or antagonism is computed by analyzing whether the effect of two compounds on this set of genes is concordant or discordant by means of a compound-pair interaction score. The advantage of the last model with respect to DIGRE is that it only requires transcriptomic data and information on the dose-response curves of single compounds, while DIGRE requires an extra step of gene-pathway analysis. The general conclusion of the study was that despite the accuracy of the predictions was not optimal, computational prediction of compound-pair activity (synergism and antagonism) is possible [59]. The exercise of collaborative challenge development by DREAM is clearly a framework which can be replicated in ecotoxicology to solve similar and related scientific questions.

### 5.2. The Prediction of Multicomponent Interactions Based on Previous Knowledge on Component-Component Interactions

**Figure 4:**
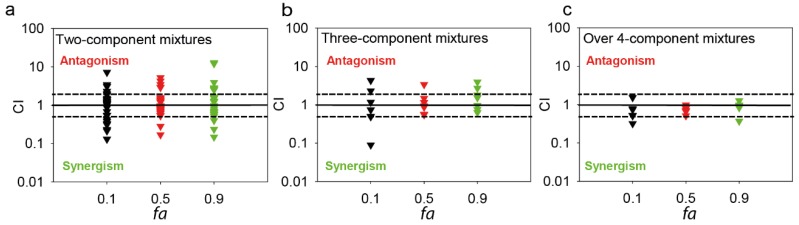
CI values of two-component mixtures (*n* = 63) (**a**); three-component mixtures (*n* = 21) (**b**) over four-component mixtures (*n* = 18), (**c**) at three representative effect levels *fa* (0.1, 0.5, 0.9 ) for a variety of pollutant combinations, such as heavy metals, herbicides, perfluorinated surfactants, and pharmaceuticals, elaborated based on the data published in Rodea-Palomares *et al.* [33,34,51], Rosal *et al.* [35] and Gonzalez-Pleiter *et al.* [38]. CI axis in logarithmic scale. Straight lines represent additivity, broken lines indicate suggested limits for significant departures from additivity (CI = 2 as upper limit for antagonism, CI = 0.5 as upper limit for synergism).

Regarding the second possibility, Warne and Hawker [62] proposed a “funnel” hypothesis postulating that, as the number of components in a mixture increases, the range of deviation from additivity decreases. Their hypothesis was validated with narcotic chemicals. A generalization of this hypothesis may imply that any increase in the number of components in a mixture may result in a reduction of departure from additivity. Classical reports on joint effects of multicomponent mixtures for acting chemicals [40,41,42] support the funnel hypothesis. Figure 4 shows a meta-analysis of the tendencies of departures from additivity as a function of the effect level (*fa*) based on our previously published data [33,34,38,51]. The figure includes departures from additivity measured as CI values for mixtures composed of different types of priority and emerging pollutants (pesticides, heavy metals, and pharmaceuticals). As can be seen in the Figure, the greatest departures from additivity are found at binary mixtures (Figure 4a). The prevalence and intensity of the departures from additivity are reduced with the increasing complexity of the mixtures (Figure 4b, c). Therefore, our data obtained using a specifically focused method on the identification of departures from additivity, seem also to support the funnel hypothesis. However, other interesting patterns can also be observed in our meta-analysis: A clear effect-level dependency of the incidence and intensity of the departure from additivity is observed: (1) the lowest incidence of departures from additivity occurred at the 50% effect level; (2) CI values tend towards one with the increasing complexity of the mixtures mainly when determined at the EC_50_ effect level. However, CI values seem to stabilize at a CI value different from one for other effect levels, especially at low effect levels (10%). In fact, the resulting CI values are near 0.5 (synergism) for some multicomponent mixtures (Figure 4c). This observation is in agreement with the observations of Laetz *et al.* [63,64] and Barran-Berdon*, et al.* [65] who found a clear effect-level dependency on the synergistic effects of tested binary combinations of pollutants.

In addition, it is interesting to note that there are some remarkable exceptions in our data regarding the decrease of the degree of the departures from additivity with the number of mixture components: some ternary mixtures are more synergistic that the corresponding binary mixtures (Figure 4a, b). These data points correspond to mixtures of pharmaceutically active substances of the family of fibrates, and mixtures of fibrates and waste-waters [34]. Similar results are not uncommon in the bibliography; for example,Cedergreen *et al.* [66], and Rosal *et al.* [35] found that ternary mixtures of antagonistic binary components resulted in increased antagonism with respect to binary mixtures. Recently, Chen *et al.* [54,55] and Wang *et al.* [36,37] found increased synergism in a series of experiments from binary to multicomponent mixtures of herbicides, pesticides, and heavy metals. These results do not seem to totally agree with the proposed “funnel” hypothesis. Cedergreen *et al.* [66] based on their own results, suggested a reformulation of the funnel hypothesis shifting the focus from the number of components to the number of possible interactions among the components in the mixture. They suggested that there were no scientific foundations to assume that adding a new synergizer to a synergistic mixture will result on a reduced synergism [66]. Interestingly, this hypothesis was clearly confirmed that same year by Tian *et al.* [67] in a thorough work focused on the factors influencing the joint effects of multicomponent mixtures including reactive chemicals. They demonstrated several interesting points: (1) a mixture of similarly acting chemicals normally resulted in additive effects (this partially explains the prevalence of additivity found in mixtures of similarly acting chemicals in the reports from the last 15 years); (2) mixtures of reactive chemicals (such as cyanogenic toxicants and aldehydes) usually results in synergism; (3) the maximal synergistic effects are found at binary mixtures, and at equitoxic ratio (the Climax theory) [68,69]; and (4) the increase in mixture complexity results in a funnel towards additivity only if the new chemical added to the mixture is not able to produce a stronger interaction than any other chemical combination already present in the mixture [67]. From our opinion, these results offer a conceptual framework to propose a revised funnel hypothesis, in which the funnel is not necessary centered on additivity (CI = 1). We propose that the multicomponent-type of interaction may converge to CI values others than one, depending on the proportion and the intensity of component-component interactions of synergistic or antagonistic pairs present in the mixture. This hypothesis is supported by the results of Tian *et al.* [67], who found that some multicomponent mixtures did not stabilize on additivity. They found some mixtures including reactive chemicals to stabilize at TUs < 0.5 even at a number of component as high as 1000. This is also in agreement with our own results [34]: when synergizers of the family of fibrates were added to a waste-water (very complex multicomponent mixture), the resulting interaction in the mixture was synergism (CIs around 0.5). In this context, the possibility of predicting the resulting type of interaction in multicomponent mixtures, that is, at which CI values the center of the “funnel” may be located, may be interesting. A first step in such an attempt is to explore possible relationships between the type and intensity of the departures from additivity occurring in low-component mixtures (for example in binary mixtures) with respect to multi-component mixtures. The pioneering work of Cedergreen *et al.* [66] proposed a method to predict ternary mixture interactions from binary mixture ones. The method was based on the idea that the highest interaction is found in binary mixtures, and that the resulting ternary mixture can be predicted using mixture ratio information and binary mixture interaction information (by fitting deviation parameters). The model described, reasonably well, ternary mixture interactions; however, the scaling of the method to an increasing number of components in the mixtures is difficult since it estimates one interaction parameter for each binary possible combination. In this regard, the idea of master binary mixture interactions dominating the multicomponent-type of interaction [34], offers a practical way forward to dispel the cost of the dimensionality associated with the estimation of all possible pair-pair interaction parameters. Tian *et al.* [67], in their thorough work on multicomponent mixtures, also proposed and found evidences supporting the idea of master binary interactions dominating the pattern of interaction in multicomponent mixtures. We found consistent evidences of master binary mixture interactions dominating ternary and higher component-type of interactions [33,34]. In addition, Li *et al.* [70], developed a theoretical framework based on the so called “fishing hypothesis” to explain the patterns found in multicomponent mixture interactions based on the ratios of the different chemicals present in the mixtures. However, for the moment, the prediction of the resulting type of interaction in multicomponent mixtures is a non-addressed challenge both in toxicology and ecotoxicology.

For the moment, the only available practical risk assessment framework allowing to integrate existing information on chemical non-additive interactions is the so called Binary Weight of Evidence (BIBWOE) developed by the US Dep. of Human Health and Services [71]. In the BIBWOE approach, information on binary mixture interactions is compiled and tested for completion. An interaction-based hazard index (HIint) is computed including a correction of additivity prediction (if required) when confident binary interaction information exists. The main limitation of the approach is the uncertainty in the translation of HIint obtained from binary mixtures to multicomponent mixtures, which is made by an *ad hoc* procedure. To our knowledge, no similar approach exists in European legislation. In fact, in the European context, it is generally argued that the incidence of departures from additivity is irrelevant in terms of risk estimation for two reasons previously discussed: (1) the supposed low incidence of synergism; and (2) the tendency to additivity of multicomponent mixtures according to the “funnel” hypothesis [6]. However, as previously discussed, Cedergreen [58] found near 30% prevalence of synergistic interactions (one of each three binary mixtures results in synergism) in other than pesticide context. In addition, there are empirical evidences of non-additive convergence of some multicomponent mixtures [34,67]. Therefore, an approach integrating available information on known synergizers may not lack interest [56,71]. Marx *et al.* [56] performed an ERA of antibiotic mixtures using the BIBWOE approach and compared its results if interactions among antibiotics are omitted. They found that the risk underestimation ranged between 50% and 200% if synergism among antibiotic families was not taken into account. These numbers are in fact equivalent to a multicomponent CI value between 0.75 and 0.5 (toxicity 1.5–2 times higher than the expected under the additivity assumption). These data, which are in agreement with our own data for multicomponent mixtures [33,34,38], seem to indicate that an assessment factor of at least two over Loewe additivity (CA) may be reasonable to account for unexpected synergistic interactions when performing risk assessment of complex mixtures of pollutants. However, as stated by Marx *et al.* [56], the uncertainty in their analysis, and therefore of its risk estimation, is presently very high due to the lack of systematic studies on interaction information on binary and multicomponent mixtures.

## 6. Fractional Approaches in Real World Conditions: Are They a Suitable Way Forward?

Fractional approaches have demonstrated to constitute a reasonable and powerful quantitative framework for the conceptual and practical analysis of chemical mixture effects under “optimal experimental conditions”. However, there is an important mismatch between real world conditions on which the conclusions of mixture ecotoxicology are asked to give answers and the optimal conditions where fractional approaches have been evaluated. In fact they are usually so far from this optimal experimental conditions that have forced a general concern on their actual applicability [2,53,72]. In this section we are going to discuss the practical and sometimes conceptual limitations that the use of fractional approaches and “optimal experimental conditions” have unnoticeably imposed on the way we consider and deal with mixture research in ecotoxicology. We are going to discuss specifically the following points: (1) chemical concentrations at or near the toxicological thresholds of observable effects; (2) mixtures of individually “active” chemicals only; (3) mixtures at limited and very specific component ratios; (4) mixtures and its potential toxicity as a static entities; and (5) mixtures evaluated on apical end-points and not considering interactions with other toxicologically relevant non-chemical factors.

### 6.1. Chemical Concentrations at or Near the Toxicological Thresholds of Observable Effects

As previously discussed, there is an overwhelming body of evidence in pharmacology, toxicology, and ecotoxicology supporting that fractional approaches and conceptual models available to sum up expected effects of chemical mixtures (CA and IA) offer reasonable quantitative expectations [1,6]. However, all those empirical evidences are presented usually at very high doses: at concentrations on the dose-response range of the individual chemicals, which are far from real world concentrations (usually several orders of magnitude). As discussed, valuable efforts have been made, however, on the applicability of the fractional approaches at low concentrations (on the NOEC region) [21,42,46]. However, even these concentrations (NOECs) are usually far from realistic exposure concentrations. In fact, the basic assumption of fraction linearity remains to be tested regarding the extrapolations from high to very low doses [72]. Evidence exists on the non-linearity of the responses below the thresholds of observable monotonic responses. The most common and accepted case is hormesis [73,74]. Observable hormetic response extends usually around an order of magnitude below the concentration of the monotonic response threshold [74]. However, even hormesis is somehow a monotonic (parabolic) observable response. There is evidence of compounds resulting in sinusoidal responses along concentrations several orders of magnitude below the threshold of monotonic observable response (including hormesis). For example, Quinn *et al.* [75] found evidence of sinusoidal presence/absence of statistically significant effects of mixtures of pharmaceuticals through dilutions of four orders of magnitude on a hydra model organism. MacMahon *et al.* [76,77] found statistically significant sinusoidal effects along a concentration range of four orders of magnitude of the fungicide chlorothalonil on the corticosterone levels, immunity, and mortality in *Rana sphenocephala* in outdoor mesocosms. Laetz *et al.* [64] found an unusual steep concentration-response relationship across a mere two-fold increase in the concentration of a mixture of two pesticides (Diazinon, Malathion) on juvenile Coho salmon which was unpredictable from their individual dose-response curves.

### 6.2. Mixtures of Individually *“*Active*”* Chemicals Only

Key experiments demonstrating the accuracy of the predictions of additivity models (CA and IA) have been performed under “optimal conditions” regarding the components present in the mixtures, usually using mixtures of pure active substances. For example, similarly active chemicals *vs.* totally dissimilarly acting chemicals [40,41,42]. From the view-point of using the best possible experimental conditions to facilitate the hypothesis testing, it is obviously totally justified. However, we should keep in mind that the results obtained are somehow biased from the view point of realism. To assume that these results are directly representative of what would be expected in real world conditions takes several non-recognized assumptions. One of these assumptions is that individually non-active chemicals (or factors) would never contribute significantly to the resulting chemical mixture effect. Considering the mathematical formulation of additivity (see Equations (1), (2) and (8)), it is clear that it is, in fact, a default assumption (or mathematical limitation) of the present definitions of additivity, since only the chemicals to which “fractions of effect” can be derived, can be included to compute mixture effect predictions. Individually non-active chemicals have, in fact, been suggested to be ignored for risk assessment purposes [78]. However, it is now clear that non-individually active chemicals can substantially modify mixture toxicity [53,79,80]. For example, Escher *et al.* [79], in a work on mixtures of 296 chemicals found that only one of 21 mixtures of individually active oxidative chemicals deviated more than two times from the predicted joint activity. In contrast, all the seven mixtures tested containing five potent and 15 non-potent (non-active) pesticides gave a consistently higher effect than that predicted by CA for the five potent compounds. In fact they found that the deviations for these seven mixtures were quite large compared to those observed for mixtures containing just potent compounds (up to four times more potent). Similarly, Frische *et al.* [81] found that non-estrogenic chemicals were able to modify substantially the estrogenic activity of individual estrogens and their mixtures. Interestingly, non-estrogenic chemicals were able to result both in synergistic effects (increased estrogenicity), and false negative (non-detected estrogenicity due to the interference of toxicity of non-estrogenic chemicals). Recently, Carvalho*, et al.* [53], found that when dubious active pharmaceuticals (diclofenac, carbamacepine, and Sulfamethoxazole) were added to a mixture of 14 priority substances at their European level quality standard concentrations, the mixture resulted in an up to 10 times increased effect. In addition, apart from departures from the expected effect of mixtures, individually non-active chemicals can totally change the expected effects of mixtures. For example, Chung*, et al.* [82] found that triclocarban (TCC), a non-xenostrogen antibacterial compound strongly enhanced the expression of estrogen in the brain of early zefrafish embryos but suppressed the estrogenic activity of bisphenol A. Jernbro *et al.* [83] found that perfluorooctane sulfonate (PFOS) alone demonstrated no genotoxicity up to a concentration of 12.5 µg/ml. However, PFOS combined with cyclophosphamide (CPP) caused a significant increase of the genotoxic potential of CPP. Adam *et al.* [84] found that a mixture of wood preservative pesticides was far more toxic when individually-innocuous solvents and additives were added to the mixture of active ingredients. Dondero *et al.* [85] found in a transcriptomic assessment that the response to individual exposure to nickel and the pesticide chlorpyrifos was qualitatively different to that produced by the exposure to the mixture of both.

### 6.3. Mixtures at Limited and Very Specific Component Ratios

Mixture ecotoxicology has focused classically in effect-oriented ratios of mixture components rather than in exposure-oriented ratios. For example, see [34,41,42]. Again, the reason for such an approach is based on the easier way from a methodological and conceptual view point. The problem with realistic mixtures is that their components are normally clearly uneven in potency, for example see [20,86]. This has two important implications: (1) real complex mixtures are more similar to a low component mixture of few chemicals mixed with a highly complex matrix. This kind of mixtures can result in strong interaction effects [34,35]; and (2) as previously discussed, non-additive effects are not uncommon in low-component mixtures [67,87]. Even trivial mixtures can result in non-expected interacting effects. For example, Charles *et al.* [88] found that simple binary mixtures of copper and nickel resulted in unexpected toxic interactions in the fresh-water amphipod *Gammarus pulex* depending just upon the applied mixture ratio.

### 6.4. Considering the Mixture and Its Potential Toxicity as a Static Entity

Dynamic aspects of mixture exposure are presently poorly considered in mixture ecotoxicology. Dynamics in mixture ecotoxicology should consider aspects regarding emission patterns, contaminants partitions, flows, transformation, and degradation. Similarly, it should consider exposure trends, in the sense that exposure may or not be coincident in time (exposure history), and may or may not be continuous [89]. Despite some fruitful works have been made regarding differential exposure and exposure history, such as the Pollution Induced Community Tolerance (PICT) [90,91], obviously, dynamics increases a layer of complexity that surely mixture ecotoxicology is presently far from solving in the predictive aspect. On the other hand, from a practical perspective it can be argued that exposure dynamics is related with the exposure aspect of risk assessment and not with the effect characterization, and therefore it does not have implications on the applicability of fractional approaches. However, the assumptions formulated by mixture ecotoxicology, such as the increasing prevalence of additivity with the number of components in the mixture (the funnel hypothesis), overlook the dynamic aspects by default. This implies that, from the classical viewpoint of mixture effect research and risk assessment, it does not matter whether no-effect comes from truly no-effect, or by an accumulation of synergistic and antagonistic effects. The emergence of Effect Directed Screening (EDS) [4,92] made clear that no observable effect is not synonym of safe. We should wonder whether we are just comfortable knowing that an specific complex mixture effect can be predicted or not by an additive model, or whether it is important if the mixture is truly additive, or contains a series of components which may potentially result in increased hazard when suffering dynamic changes [93].

### 6.5. Mixtures Evaluated on Apical End-Points and Not Considering Interactions with Other Toxicologically Relevant Non-Chemical Factors

Since ecotoxicology derived from human toxicology, the focus is historically posed on the organismal/population level. Additivity hypothesis has been tested mainly on apical end-points (such as mortality, growth inhibition, or apical metabolic activities). However empirical evidence is required to elucidate the applicability of fractional approaches and present formulations of additivity on both sub-organismal and supra-organismal end-points [20]. At the sub-organismal level of organization (for example, at the level of gene expression or protein synthesis) [94], a general problem for the definition of additivity is that the end-points are usually bi-directional: they can be up-regulated and down-regulated (or both, depending on concentration) in response to chemical exposure, and differentially-expressed genes can emerge from single to combined exposures [95]. Since the biological responses violate the basic requirement of monotonicity of the dose-response curves, it is impossible to define a homogeneous and ever-holding fractional effect scale,and, therefore, additivity equations cannot be solved [96]. In addition, additivity would be computed in any of hundreds or thousands of expressed genes or proteins which may result in synergism/antagonism for the same chemical depending on the considered activity. The work of Bansal*, et al.* [59] opens a totally novel and sound conceptual and practical framework for mixture research in a toxico-transcriptomic context (see Section 5). Equivalently, the definition and applicability of fractional additivity formulations has not been validated for some interesting and powerful emerging end-points such as behavioral end-points (predator avoidance, sociality, territoriality, *etc.*) [97,98].

Regarding the supra-organismal level of organization, the problem is similar in the sense that the definition of dose-response patterns and fractional effects may not be an operative way to define additivity, since many effects are driven and propagated indirectly along the system’s architecture. Despite this, the only available practical approaches to predict risk for species assemblages are built upon single specie-derived Species Sensitivity Distributions (SSDs) and classical additivity models (CA, IA, or a combination of both [99]). These kinds of approaches, despite being valuable and built upon the best information presently available ignored, by default, any biotic and abiotic interactions and indirect effects. However, it is becoming clear that indirect effects are an extremely important factor in real world systems. Liess *et al.* [100] found that the populations of an aquatic invertebrate (*Culex pipiens*) exposed over several generations to repeated pulses of low concentrations of thiacloprid declined and did not recover in the presence of a less sensitive competing species (*Daphnia magna*). However, in the absence of a competitor, insecticide effects on the more sensitive species were only observed at concentrations one order of magnitude higher. In a study on macrocosms developed by Rohr *et al.* [48], the interplay of atrazine and trematodes leads to the increased mortality of amphibians via a complex network of indirect effects along the ecosystem. However atrazine *per se* had poor effects on the amphibian itself. In addition, trematodes-increased infection depended indirectly upon atrazine, since it relied upon the increased proliferation of snails which, in turn, depended on the tolerance of algae to atrazine with respect to aquatic plants [48]. It is clear that an additivity definition based on a fractional approach is way too simple for being applicable in this case. In fact, in such a complex relationship ecotoxicology is basically unable to predict even the resulting effect (not just if it is or not additive). Similar considerations hold for the specific contribution of chemical mixtures in the global picture of stressors (natural and anthropogenic) to which natural populations and ecosystems are subjected. There is a basic lack of understanding and approaches to integrate chemical stressors in the global ecological picture. Some interesting works are presently trying to validate at field regional scales the predictions of risk estimated based on mixture ERA [101,102,103] and to derived chemical pressure contribution to the overall environmental effects observed at regional scale from field data [102,104]. Results are at present confusing and require further research. Some authors [101,103] found good correlations between the total sum of TUs (based on the CA model) estimated from laboratory experiments and biodiversity loses and traits indicators (SPEARs) at the regional scale in the field. However, actual effects occurred at concentrations even 1000 times lower than those expected based on laboratory-derived Species Sensitivity Distributions (SSDs) (therefore, interactions among stressors seem to change only in terms of potency the expected effects of chemical pressure). Other authors [102] found that the relevance of interactions among factors are so important that to consider only the influence of single predictors (including chemical pressure) may lead to biased interpretations of the relevance of the different factors on the actual environmental impact. Laskowski *et al.* [105] in a meta-analysis based on 61 different studies on effects of temperature, moisture, and dissolved oxygen on the toxicity of a range of chemicals including heavy metals, pesticides, and polycyclic aromatic hydrocarbons found that, statistically significant interactions occurred between natural factors and chemicals in the 62% of cases. In addition, the significant interactions included second-order interactions, indicating that not only the single chemical toxicity, but the nature of the interaction among chemicals can be modified by natural conditions. There are several interesting revisions on the interacting effects of chemical pollution and natural environmental factors [80,105,106,107]. As well as interactions of chemical pollution and climate change [108,109]. Conclusions are worrying: An increasing agreement exists on the need of adopting an ecosystem perspective in ecotoxicology including mixture ecotoxicology, and the need for the implementation and adaptation of new tools, models, and analysis to predict chemical effects on biodiversity in real-world conditions [3,89,110,111]. Relevant open questions are whether fractional approaches are the adequate frameworks to go forward, and if they will be able to assume the challenge of demanded increased levels of complexity and realism in the future ecosystems-oriented ecotoxicology.

## 7. Conclusions

The present critical review has summarized the available and commonly used fractional approaches to study/predict mixture effects (additivity, synergism, and antagonism) in pharmacology and ecotoxicology (CA, IA, and CI). We summarized the origin of the concepts, parallel evolution, and their contribution in the progress of mixture ecotoxicology in the last 20 years. In our opinion, strengths and weaknesses of the additive mixture models (CA and IA) come from their basic assumption of additivity between mixture components. This assumption has demonstrated to offer a robust and reasonable estimation of the combined effect of chemical mixtures. However, the assumption of additivity ignores potential interactions such as synergism and antagonism between mixture components. In this regards, Combination Index (CI) offers a framework where interactions are not ignored. However, for a predictive formulation, a parameter on the interactions among mixture components should be estimated (predicted). Despite the prediction of toxicological interactions is still not satisfactorily addressed in pharmacology and/or ecotoxicology, we discuss the possibility of predicting interactions among mixture components at two levels: interactions among pairs of chemicals, and multicomponent interactions. We summarized the existing initiatives, and we found consistent evidences supporting the possibility of developing quantitative frameworks for both levels. Finally, we discussed the open questions and limitations of fractional approaches in the context of increased realism as a main future challenge for the 21th Century ecotoxicology (including mixture ecotoxicology).
